# Epstein–Barr Virus-Induced Post-Transplant Lymphoproliferative Disorder of the Central Nervous System Successfully Treated with Chemo-Immunotherapy

**DOI:** 10.3390/v12040416

**Published:** 2020-04-08

**Authors:** Hiroaki Inoue, Shinya Rai, Hirokazu Tanaka, J. Luis Espinoza, Maiko Komori-Inoue, Hiroaki Kakutani, Shuji Minamoto, Takahiro Kumode, Shoko Nakayama, Yasuhiro Taniguchi, Yasuyoshi Morita, Takeshi Okuda, Yoichi Tatsumi, Takashi Ashida, Itaru Matsumura

**Affiliations:** 1 Department of Hematology and Rheumatology, Kindai University Faculty of Medicine, Osaka 589-8511, Japan; h.inoue@med.kindai.ac.jp (H.I.); htanaka@med.kindai.ac.jp (H.T.); luis@staff.kanazawa-u.ac.jp (J.L.E.); komomai@med.kindai.ac.jp (M.K.-I.); jamine311@hotmail.co.jp (H.K.); 2710mshuji@gmail.com (S.M.); kumode@med.kindai.ac.jp (T.K.); s.nakayama@med.kindai.ac.jp (S.N.); m11049@med.kindai.ac.jp (Y.T.); moriyasu@med.kindai.ac.jp (Y.M.); anzen2@med.kindai.ac.jp (Y.T.); ashida@med.kindai.ac.jp (T.A.); i.matsu@med.kindai.ac.jp (I.M.); 2 Department of Neurosurgery, Kindai University Faculty of Medicine, Osaka 589-8511, Japan; okuda@med.kindai.ac.jp

**Keywords:** aplastic anemia, EBV, lymphoproliferative disorder, immunosuppressive therapy, transplant complications

## Abstract

Aplastic anemia is a rare blood disease characterized by the destruction of the hematopoietic stem cells (HSC) in the bone marrow that, in the majority of cases, is caused by an autoimmune reaction. Patients with aplastic anemia are treated with immunosuppressive drugs and some of them, especially younger individuals with a donor available, can be successfully treated with hematopoietic stem cell transplantation (HSCT). We report here a rare case of post-transplant lymphoproliferative disorder (PTLD) associated with Epstein–Barr virus (EBV) reactivation in a 30-year-old female patient who underwent allogeneic HSCT for severe aplastic anemia. The PTLD, which was diagnosed 230 days after transplantation, was localized exclusively in the central nervous system (specifically in the choroid plexus) and manifested with obvious signs of intracranial hypertension. After receiving three cycles of high dose methotrexate (HD-MTX) combined with rituximab, the patient achieved a complete clinical recovery with normalization of blood cell counts, no evidence of EBV reactivation, and no associated neurotoxicity.

## 1. Introduction

Aplastic anemia is a rare and serious blood disease characterized by a failure of the bone marrow to produce mature blood cells. In the majority of cases, this condition is caused by an autoimmune disorder in which the immune system, mainly T-cells, mistakenly attacks and destroys hematopoietic stem cells in the bone marrow leading to pancytopenia and bone marrow hypoplasia [1,2].

In general, patients with aplastic anemia are treated with long-term immunosuppressive therapies, including cyclosporine-A and anti-thymocyte globulin. However, for young patients with an available donor, hematopoietic stem cell transplantation (HSCT) is the preferred therapeutic approach [3]. Although, if successful, HSCT can cure aplastic anemia, this therapy is also associated with severe complications, including graft versus host disease (GVHD), increased risk of severe infections, and the development of lymphoproliferative disorder (LPD), particularly post-transplant lymphoproliferative disorder (PTLD) [4], which is characterized by the excessive and uncontrolled proliferation of lymphocytes (mostly B cells) as a consequence of induced immunosuppression after solid organ or HSCT [5,6].

The clinical and pathological spectrum of PTLD is variable, ranging from reactive hyperplasia to malignant lymphoma (ML), including diffuse large B-cell lymphoma (DLBCL), peripheral T-cell lymphoma (PTCL), and classic Hodgkin lymphoma (CHL). Etiologically, the majority of PTLD cases are caused by the reactivation of Epstein–Barr virus (EBV) infection, as a result of the intense immunosuppressive therapy given to these patients [7].

EBV is a ubiquitous microorganism that infects more than 95% of the adult population worldwide. The virus is etiologically linked to several human cancers. These include CHL, Burkitt’s lymphoma, DLBCL, malignant lymphomas of T or NK cells and other tumors arising from epithelial tissues, including nasopharyngeal carcinoma, some subsets of gastric carcinoma, and leiomyosarcoma [8,9].

EBV infection is usually acquired during early childhood, where it can be asymptomatic or may cause a transient febrile disease with flu-like symptoms. In some individuals, especially when the infection is acquired during adolescence, the virus can cause infectious mononucleosis syndrome. In immunocompetent hosts, the virus is successfully cleared by the immune system, but it persists as a latent infection in memory B cells [8,9]. The dormant virus reactivates in immunocompromised individuals where the impaired immune response, mediated by T cells, fails to control the proliferation of EBV-infected B cells, leading to the development of LPD and PTLD [7].

In general, PTLD is a systemic disease, although localized variants are also common, which can affect almost any site of the body including the gastrointestinal tract, the lungs, and the liver [10]. The involvement of the central nervous system (CNS) is uncommon and can occur either as an extension of a systemic PTLD, or as the primary, or the only, affected site, and is always associated with a dismal prognosis, which is further complicated by the fact that treatment guidelines for this entity have not been established [11]. Pathologically, primary lymphomas arising in the CNS, which are typically DLBCL, frequently affect the following sites: the frontal lobes, temporal lobes, and parietal lobes, followed by the basal ganglia and periventricular brain parenchyma. Primary lymphomas originating from the choroid plexus are extremely rare [12].

The current treatment of PTLDs includes the tapering of immunosuppression (reducing the dose of the immunosuppressive agents) combined with immunotherapy with anti-CD20 monoclonal antibodies to target lymphoma cells, which in most cases express the CD20 receptor on their surface [5]. In addition, chemotherapy regimens, often those including high-dose methotrexate (HD-MTX) combined with rituximab and other cytostatic drugs that penetrate the blood-brain barrier, have also been employed [5,13]. Moreover, patients who achieve a complete response to chemotherapy often receive whole-brain radiotherapy (WBRT) for consolidation. However, despite the high response rate of WBRT, this approach is associated with a high risk of severe neurotoxicity and should be reserved for patients who do not qualify for other systemic treatments [14].

Here, we report a rare case of CNS-PTLD occurring within the first year after HSCT, which originated from the choroid plexus. This case was diagnosed by surgical biopsy and was successfully treated with HD-MTX and rituximab without neurotoxicity.

## 2. Materials and Methods

### 2.1. Patient Consent

Blood human samples were taken after obtaining the informed consent from the patient in accordance with the tenets of the Declaration of Helsinki.

### 2.2. RT-PCR for Assessing EBV Load

For the PCR assay, the DNA was extracted from the blood plasma and cerebrospinal fluid (CSF) fraction by using a QIAamp Blood Kit (QIAGEN Inc., Chatsworth, Calif, USA). The CSF levels and plasma levels of EBV DNA were determined with the quantitative PCR method, using the primers which were designed as previously reported [15]. To generate EBV positive control, the BALF5 gene of EBV was cloned into the pGEM-T vector (Promega, Madison, WI, USA) to generate pGEM-BALF5, and serial dilutions of this construct were utilized as template DNA in the real-time quantitative PCR reactions to generate a standard curve. The CT values from clinical samples were plotted on the standard curve, and the copy number was calculated automatically by Sequence Detector version 1.6 software package (PE Applied Biosystems, Waltham, MA, USA).

### 2.3. Tissue Processing, Staining, and Immunohistochemistry

Tumor tissues were fixed in 10% buffered formalin and embedded in paraffin. Sections were cut to a thickness of 4 µm and stained with hematoxylin and eosin (H-E). Immunohistochemical stains were performed with a Ventana BenchMark XT automated staining instrument (Roche Tissue Diagnostics, Tokyo, Japan), according to the manufacturer’s protocol, with the following primary antibodies: anti-CD20 (L26, Roche Diagnostics K.K., Tokyo, Japan), anti-CD79a (JCB117, Nichirei, Tokyo, Japan), anti-BCL2 (124, Agilent Technologies, Santa Clara, CA, USA), and anti-MUM1 (mum1p, Agilent Technologies). In situ hybridization for Epstein–Barr virus-encoded small RNAs (EBER) was performed on FFPE (formalin-fixed paraffin-embedded)tissue section using a (FITC fluorescein isothiocyanate) labeled oligonucleotide probe (Roche Diagnostics K.K.) supplied by Ventana on an automated staining instrument (Ventana-Benchmark, Tucson, AZ, USA).

### 2.4. Serological Studies

Serum samples were tested for anti-EBV-VCA-IgM, anti-EBV-VCA IgG, anti-EBNA-1 IgG, and anti EBV-EA-D IgG antibodies with an immunofluorescence test (BML Inc., Tokyo, Japan). Positivity was considered when anti-EBV-VCA-IgM (≧10), anti-EBV-VCA IgG (≧10), anti-EBNA-1 IgG (≧10), and anti-EBV-EA-D IgG (≧10).

## 3. Case Presentation

**Figure 1:**
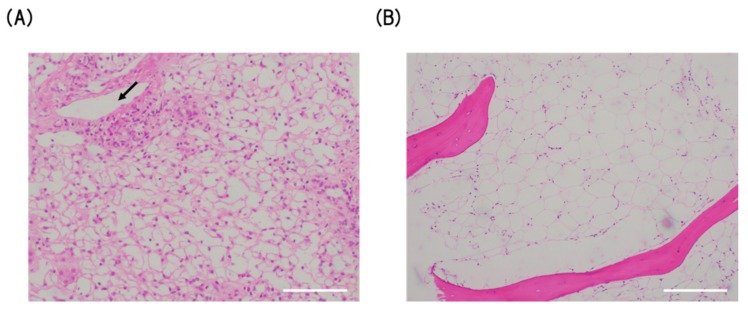
(**A**) Liver biopsy showed periportal zonal necrosis with macrovesicular steatosis compatible with the diagnosis of acute hepatitis (H-E (hematoxylin and eosin) staining, ×10). Arrow indicates portal vein. (**B**) Bone marrow biopsy showed decreased hematopoietic cells (hypocellular marrow), with increased adipose tissue consistent with the diagnosis of aplastic anemia (H-E staining, ×10). Scale bar, 500 µm.

A previously healthy 30-year-old female was referred to our hospital in January 2016 for the assessment of jaundice associated with loss of appetite. Laboratory studies revealed a bilirubin level of 14.0 mg/dL (normal level 0 to 0.4 mg/dL), an alanine aminotransferase (ALT) level of 1655 U/L (7 to 23 U/L), and an aspartate aminotransferase (AST) level of 1750 U/L (13 to 30 U/L), consistent with the diagnosis of acute hepatitis. Further serologic studies showed no evidence of a viral infection, with negative tests for hepatitis A, B, C, D, and E viruses. Antibodies against cytomegalovirus (CMV), including anti-CMV-immunoglobulin (Ig)M and anti-CMV-DNA, were also negative. Antibodies against EBV, including anti-EBV-VCA IgG (20) and anti-EBNA-1 IgG (10), were positive, whereas anti-EBV-VCA-IgM (<10) and anti EBV-EA-D IgG (<10) were negative, consistent with prior but not active EBV infection. Parvovirus B19 DNA was also negative in the serum. Antibodies to anti-liver-kidney microsome, anti-smooth muscle, and anti-nuclear were also negative, and the drug screen of urine and serum was negative. A transjugular liver biopsy showed evidence of severe acute hepatitis with bridging submassive necrosis (Figure 1A). She was therefore treated with oral prednisolone (1 mg/kg) for non-infectious severe acute hepatitis.

Within approximately one month of treatment, improvements in the clinical features were observed, with ALT and AST levels returning to normal values. However, three months after the diagnosis, rapid and progressive pancytopenia associated with symptoms of anemia (weakness, pale skin, and shortness of breath) were observed, which required the patient to be hospitalized. Laboratory findings revealed pancytopenia with an absolute neutrophil count of 300/μL, a reticulocyte count of 8920 cells/dL, and a platelet count of 56,000/μL in the peripheral blood.

A bone marrow aspirate and biopsy showed severely hypoplastic marrow with no evidence of malignancy (absence of blast cells, dysplasia, or atypical cells) and increased adipose tissue (Figure 1B). A chromosome analysis in 20 metaphases showed the absence of abnormalities and thus a diagnosis of hepatitis-associated severe aplastic anemia was made.

Because the patient had a sibling donor whose HLA (human leukocyte antigen) was mismatched at the DR locus, we directly performed allogeneic HSCT (allo-HSCT), with peripheral blood as a stem cell source, using a reduced-intensity conditioning regimen consisting of cyclophosphamide (60 mg/kg × 2 days), fludarabine (30 mg/m^2^ × 6 days) and rabbit ATG (2.5 mg/kg for 2 days) in May 2016. Chronic GVHD was observed in the patient’s gut (grade 3) on day 120 after allo-HSCT, for which she was initially treated with prednisolone (1 mg/kg/day) in an attempt to control the GVHD. Within approximately one week, improvements in the clinical features were observed, and prednisolone could be tapered to 12.5 mg/day with 5 mg of FK506 without GVHD recurrence.

**Figure 2:**
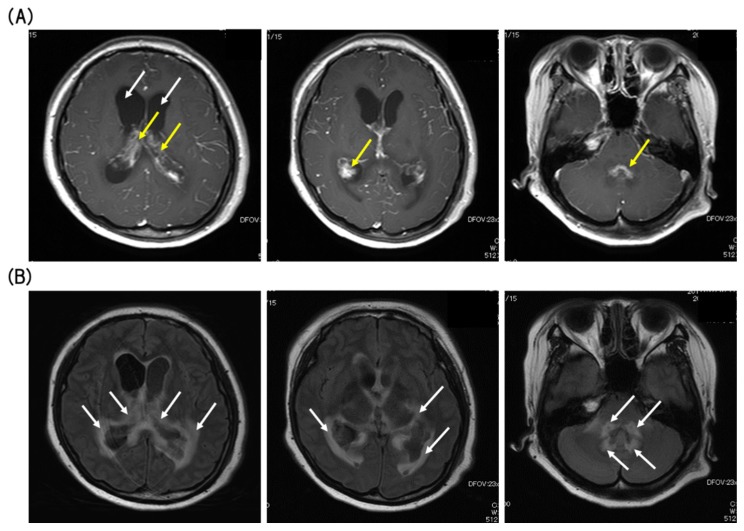
(**A**) T1-weighted (T1W) axial gadolinium-enhanced magnetic resonance imaging (MRI) showed Bilateral ventricle enlargement (white arrows), and hypertrophy choroid plexus (yellow arrows) with heterogeneous enhancement (axial sections). (**B**) Fluid-attenuated inversion recovery image (FLAIR) revealed high-intensity periventricular white-matter changes (white arrows) (axial sections).

On day 230 after transplantation, she suddenly arrived at the emergency room of our hospital presenting with severe headache, nausea, and vomiting without B symptoms (fever, night sweats, and body weight loss). Gadolinium-enhanced magnetic resonance imaging (MRI) studies of the brain demonstrated lesions in both lateral ventricles with heterogeneous enhancement, on which the lesions appeared to arise from the choroid plexus. Bilateral ventricles were dilated, and the margins of the ventricles were enhanced by contrast-enhanced fluid-attenuated inversion recovery (FLAIR)-MRI (Figure 2).

**Figure 3:**
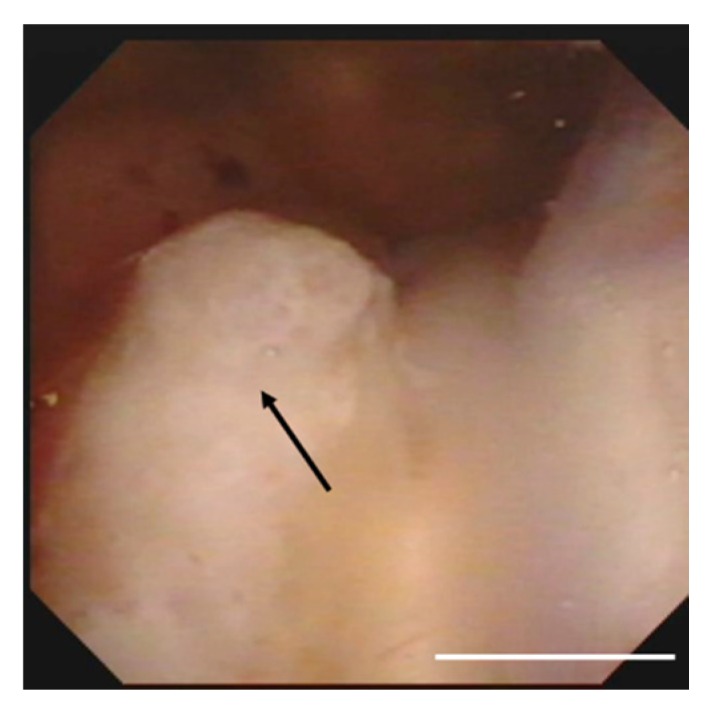
Operative findings showing a solid tumor (arrows) in the right lateral ventricle densely adherent to the choroid plexus. Scale bar, 1 cm.

Total body imaging and bone marrow aspirate histology displayed no evidence of systemic disease. Lumbar puncture was performed, and CSF analysis showed atypical lymphocyte infiltration. Microbiological studies for bacterial, mycobacterial or fungal infections were negative. On day 5, she was in altered sensorium, drowsy and disoriented, and this manifested in a loss of consciousness on day eight. A disturbance of consciousness occurred due to the progression of hydrocephalus, for which a ventricular drainage was performed. Ventricular inspection using neuro endoscopy identified the presence of a tumoral mass in the lateral ventricle, and a biopsy was taken for diagnosis (Figure 3).

**Figure 4:**
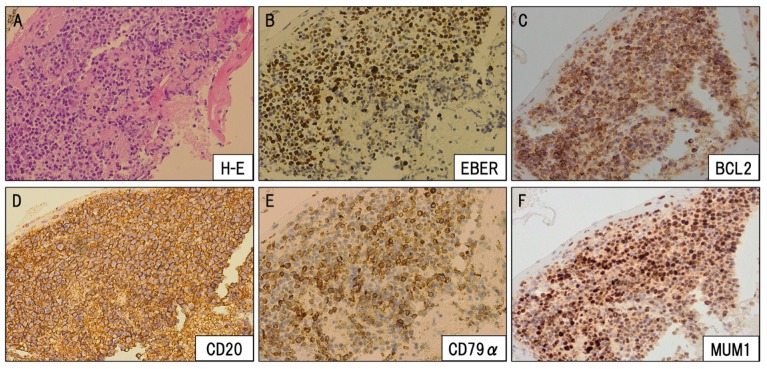
The pathologic examination of the choroid plexus region showed diffuse proliferation of large-sized lymphoid cells. (**A**) H-E staining, (**B**) Epstein–Barr virus (EBER), (**C**) BCL2, (**D**) CD20, (**E**) CD79α, (**F**) MUM1 (×10).

The biopsy showed that the tumor originated from the choroid plexus and displayed morphological features that suggested a diagnosis of monomorphic PTLD with large atypical cell proliferation. Immunohistochemistry studies revealed that those large-sized atypical cells were positive for CD20, CD79a, BCL2, MUM1, and also for Epstein–Barr virus (EBV)-encoded small RNAs (Figure 4).

RT-PCR studies for EBV showed high titers of the virus that were detectable in the CSF (3.22 × 10^5^ copies/mL) and also in blood plasma (1.97 × 10^3^ copies/mL) and thus, a diagnosis of EBV-related primary central nervous system PTLD (CNS-PTLD) was made. The immunosuppressive drug tacrolimus was stopped, and prednisolone was tapered from 25 mg to 5 mg immediately after the diagnosis, however, CT scans of the brain showed signs compatible with disease progression. The patient was then treated with immune-chemotherapy consisting of HD-methotrexate combined with rituximab (R-HD-MTX).

**Figure 5:**
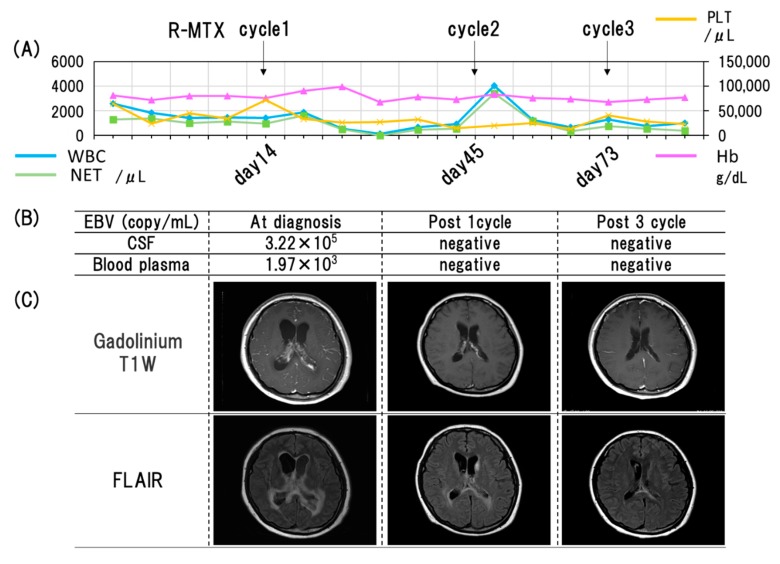
(**A**) The clinical course of this case. The X axis indicates the time (days). WBC (white blood cells), Hb (hemoglobin), PLT (platelets), NET (neutrophils), R (Rituximab), MTX (Methotrexate). (**B**) The detection of Epstein–Barr virus (EBV) by PCR analysis from cerebrospinal fluid and blood plasma and (**C**) MRI findings at the indicated period.

A dramatic clinical response was observed after three cycles of R-HD-MTX which resulted in complete disease remission, as confirmed by MRI images. Molecular studies also showed that EBV was undetectable in the CSF and in blood plasma (Figure 5). After three years of follow-up, the patient has completely recovered from aplastic anemia and there is no evidence of neurotoxicity or any sequelae of the CNS-PTLD.

## 4. Discussion

We presented here a case of primary CNS-PTLD caused by the reactivation of EBV infection in a patient receiving HSCT for aplastic anemia. The virus reactivation occurred as a result of the immunodeficient status of the patient, which was induced by the immunosuppressive drugs that were used as part of the treatment for HSCT. Distinctive characteristics of the case presented here include the development of the PTLD in the choroid plexus, the absence of B symptoms after EBV reactivation, and the excellent clinical response to chemo-immunotherapy, consisting of HD-MTX combined with Rituximab.

The choroid plexus is a lobulated structure found in the walls of the ventricles, that produces CSF and contributes to maintaining the blood-CSF barrier. It also secretes soluble factors that guide neural tissue development and contain stem cells [16]. Tumors arising from the choroid plexus are extremely rare, accounting for less than 1% of all brain tumors. The most common types of choroid plexus tumors are papilloma and carcinoma. There are a few reports of choroid plexus lymphoma; however, most of them developed in the context of a secondary involvement from pachymeningeal or leptomeningeal lesions, because choroid plexus consists of many capillaries and choroidal epithelium and might serve as the portal of entry into the CSF for disseminated lymphoma cells [17,18,19].

In addition, the choroid plexus is an important site for the initial dissemination of infectious agents capable of infecting the central nervous system, such as mycobacterium tuberculosis, cytomegalovirus, Cryptococcus, and various pathogenic bacteria [20]. Therefore, a rigorous assessment of the clinical and laboratory data is essential for the accurate diagnosis of the local lesions affecting the choroid plexus.

Clinical features of patients with choroid plexus lymphoma are nonspecific, and thus, MRI images play a crucial role in the disease diagnosis and are essential for monitoring the response to treatment. In most cases, primary choroid plexus lymphomas are observed as enhanced mass lesions on MRI scans without hydrocephalus [17,21]. Because hydrocephalus and the margins of the ventricles were enhanced by FLAIR-MRI in the presented case, we could not rule out the possibility that the lesions were related to various types of infections. Therefore, the final diagnosis was made by the surgical biopsy.

In general, PTLD frequently presents in the first year after transplantation, especially in EBV-seronegative recipients who acquire early post-transplant EBV infection. In CNS-PTLD, it has been reported that time from transplantation to CNS-PTLD can be more than 10 years, and most CNS-PTLDs are associated with latent EBV infection [10]. In the case presented here, in addition to the profound immunosuppression associated with HSCT that triggered the reactivation of EBV infection and ultimately the malignant transformation of B cells, the patient was also subject to other factors, including the transplantation from an HLA-mismatched donor and the use of anti-thymocyte globulin that, based on previous reports, appear to increase the risk of PTLD [10,22].

Treatment of PTLD has been importantly strengthened by Rituximab, a humanized anti-CD20 antibody, which has been widely approved against CD20 positive malignant lymphoma including DLBCL [23]. Although Rituximab has been well established, and is an essential part of the treatment of systemic PTLD, the efficacy of Rituximab for CNS-lymphoma or CNS-PTLD still needs to be debated, because Rituximab has poor CNS penetration due to its large size [24]. Nevertheless, some case reports suggest that, despite the limited concentration of the agent in the CNS, the drug has activity in this pharmacologic sanctuary. In the presented case, EBV copies were detectable by PCR both in CSF and in blood plasma; thus, we added Rituximab to HD-MTX, not only as induction therapy for CNS-PTLD, but also as a preemptive therapy to prevent the systemic PTLD development [25].

The blood-brain barrier (BBB) is a selective semipermeable border formed by the tight junctions between the endothelial cells of brain capillaries, that restricts the passage of solutes and large or hydrophilic molecules into the cerebrospinal fluid, while allowing the transit of hydrophobic and small polar molecules. As a result, the arsenal of agents used for the treatment of CNS neoplasms is limited to those therapeutic molecules capable of passing the BBB [26]. Consequently, large molecules such as therapeutic monoclonal antibodies, including Rituximab, may transit poorly through the normal BBB, which could limit the effectiveness of this agent in PCNSL. Nevertheless, rituximab concentrations in CSF after its IV administration vary largely among patients tested, and its level correlates with the integrity of the BBB [27], where a disruption in the BBB results in enhanced rituximab penetration in brain tumor tissues [28]. In the case presented here, it is plausible that increased permeability of the BBB, likely induced by inflammation associated with EBV reactivation or the tumor itself, may have contributed to enhancing the penetration of Rituximab into the brain, which ultimately resulted in an excellent therapeutic response.

Methotrexate, an antifolate and antimetabolite given intravenously as high dose chemotherapy, is currently regarded as the most important and beneficial drug for CNS lymphoma and CNS-LPD. It has also been reported that the combination of HD-MTX with various types of drugs with known abilities to cross the blood–brain barrier, such as high-dose cytarabine, improves the response rate and progression-free survival; however, no significant increase in overall survival was noted [29,30]. Therefore, in the case presented here, we selected HD-MTX without a combination of high-dose cytarabine.

The appropriate number of HD-MTX therapy cycles needed is currently unknown. A minimum of four to six injections is delivered in most chemotherapy regimens, especially if no consolidation treatment is scheduled in the protocol [31]. In the present case, due to the dramatic improvement of the clinical symptoms and complete remission in accordance with the disappearance of EBV both in CSF and blood plasma, we utilized only three cycles of HD-MTX and Rituximab.

Recent data suggest that EBV viral load monitoring appears to correlate better with treatment response than monitoring in the cellular compartment. However, it has still been controversial with regard to whether viral load monitoring after clearance can predict disease relapse or not [32]. Moreover, there is a report that adult PTLD patients have been observed to relapse, even in the presence of a persistently low viral load [33]. Thus, potential EBV DNA reactivation and clinical features should be carefully monitored.

To our knowledge, this is the first case of primary CNS-PTLD originating from the choroid plexus after HSCT for aplastic anemia that has been successfully treated with HD-MTX and Rituximab.
